# Iodine as a potential endocrine disruptor—a role of oxidative stress

**DOI:** 10.1007/s12020-022-03107-7

**Published:** 2022-06-20

**Authors:** Małgorzata Karbownik-Lewińska, Jan Stępniak, Paulina Iwan, Andrzej Lewiński

**Affiliations:** 1 Department of Oncological Endocrinology, Medical University of Lodz, 90-752 Lodz, Poland; 2 Polish Mother’s Memorial Hospital-Research Institute, 93-338 Lodz, Poland; 3 Department of Endocrinology and Metabolic Diseases, Medical University of Lodz, 93-338 Lodz, Poland

**Keywords:** Iodine, Endocrine disruptor, Thyroid, Oxidative stress, Free radical

## Abstract

**Purpose:**

Iodine is an essential micronutrient required for thyroid hormone biosynthesis. However, overtreatment with iodine can unfavorably affect thyroid physiology. The aim of this review is to present the evidence that iodine—when in excess—can interfere with thyroid hormone synthesis and, therefore, can act as a potential endocrine-disrupting chemical (EDC), and that this action, as well as other abnormalities in the thyroid, occurs—at least partially—via oxidative stress.

**Methods:**

We reviewed published studies on iodine as a potential EDC, with particular emphasis on the phenomenon of oxidative stress.

**Results:**

This paper summarizes current knowledge on iodine excess in the context of its properties as an EDC and its effects on oxidative processes.

**Conclusion:**

Iodine does fulfill the criteria of an EDC because it is an exogenous chemical that interferes—when in excess—with thyroid hormone synthesis. However, this statement cannot change general rules regarding iodine supply, which means that iodine deficiency should be still eliminated worldwide and, at the same time, iodine excess should be avoided. Universal awareness that iodine is a potential EDC would make consumers more careful regarding their diet and what they supplement in tablets, and—what is of great importance—it would make caregivers choose iodine-containing medications (or other chemicals) more prudently. It should be stressed that compared to iodine deficiency, iodine in excess (acting either as a potential EDC or via other mechanisms) is much less harmful in such a sense that it affects only a small percentage of sensitive individuals, whereas the former affects whole populations; therefore, it causes endemic consequences.

## Endocrine disruptors

### Endocrine disruptors—general information

According to The Endocrine Society definition, an endocrine-disrupting chemical or an endocrine-disrupting compound (EDC), commonly called an endocrine disruptor, is an exogenous chemical, or a mixture of chemicals, that interferes with any aspect of hormone action [1]. This definition was proposed in 2012 [1] and it does constitute a revised and a simplified version of definitions previously recommended by various worldwide agencies such as the United States Environmental Protection Agency [2]. European Union [3], or World Health Organization [4]. It must be emphasized that this definition [1], unlike the previous ones [2–4], does not take into account whether or not EDC interference have specific adverse outcome. This is due to the fact that in many cases the harmful effects of exposure to EDCs are difficult to be fully characterized as they may not be immediately apparent and may exhibit delayed or even multigenerational effects [5]. Therefore, any interference of EDC with hormone action should be considered as a clear predictor of adverse outcome (much like mutagenicity is a predictor of carcinogenicity) [1].

It should be mentioned that some hormones (or their metabolites), especially sex hormones, i.e., androgens, estrogens, and progesterone, but also glucocorticoids, when occurring in environment in amounts exceeding these that are safe for health, may also serve as EDCs [6].

Interference caused by an EDC, mentioned in the above definition, can be related—but are not limited—to the synthesis, release, transport, metabolism, binding action, elimination of natural hormone, or binding to its receptor [7]. EDCs may bind either to orthosteric site of hormone receptors and—in this way—may exert direct agonistic or antagonistic actions or to allosteric sites modulating activity through conformational changes in the receptor producing unexpected effects [8]. However, EDC-induced interference itself does not imply a significant risk, but the real risk depends on the concentration and potency of the interacting chemical, the duration of exposure, the developmental age at which this exposure occurs, and also on individual sensitivity [9]. It should be stressed that to precisely define a threshold dose of adversity for a given EDC is not only difficult but just impossible [10].

As the endocrine system has evolved to respond to comparatively low levels of hormones—typically in the picomolar to nanomolar range—EDCs that mimic natural hormones are also likely to cause (or rather to disrupt) biological effects at low serum concentrations. In agreement with this assumption, epidemiological studies revealed links between low concentrations of EDCs and the disease prevalence [6]. However, it should be emphasized that effects exerted by low doses of EDCs are not predictable by effects at higher doses, which is due to the fact that there is a nonlinear relationship between dose and effect of EDCs (and also natural hormones) [11]. Expectedly, an association between various diseases and exposure to large concentrations of EDCs has been observed in individuals with occupational exposures [12] or after industrial accidents [13].

Particularly vulnerable are organisms at developmental age at which exposure to EDCs occurs, producing different effects at different life stages. Hormone disruption during development can produce effects that have lifetime consequences, some of which do not become manifested until adulthood [14]. EDCs impact unfavorably human reproductive health [15, 16]. Importantly, exposure to EDCs resulted in increased oxidative stress in the mother and neonate [17].

Perhaps the best known and the most common EDC is bisphenol A, which is considered to be a selective estrogen receptor modulator, revealing unfavorable effects on reproduction [1, 9]. With respect to the topic of the current review, it should be mentioned that bisphenol A exposure induces oxidative damage to membrane lipids in rats and mice, while inhibiting antioxidative defense [18].

Most EDCs come from industrial activities and encompass a wide array of agents used as pesticides, pharmaceutical agents, and additives to plastics utilized in clothing, toys, or package materials; they can also be naturally occurring agents, such as natural animal hormones or compounds produced by plants (phytoestrogens) and released into the environment, and certain fungi [19].

Certain micronutrients, mostly being non-indispensable, but also indispensable, are classified as EDCs. Regarding the function of the thyroid gland, two exogenous micronutrients, i.e., iodine and iron, are indispensable for thyroid hormone synthesis, but they can be toxic in excessive amounts [9, 20–22]. However, until now none of them is unequivocally classified as EDC.

Initially, scientists have concentrated on EDCs acting via the so-called EATS (estrogen, androgen, thyroid, and steroidogenesis) pathways and, more recently, attention has been drawn to chemicals that exert effects also through non-EATS modalities [23].

To summarize, EDCs can exert very complex actions related to several characteristics of endocrine systems. Normal hormone actions are tissue specific; therefore, EDCs may directly influence hormone action only in these tissues. In addition, tissue-specific metabolism of EDCs can lead to the formation of compounds that interfere with hormone action, primarily in tissues where they are generated. Some of the observed health effects associated with EDCs include—but are not limited—to reproductive, developmental, immunological, and neurological disorders. It should be stressed that, in opposite to experimental conditions, each living organism is exposed not to one but to numerous EDCs at any time interval [1, 9, 24]. Therefore, results of in vitro studies on EDCs should not be directly extrapolated to human organisms.

### Thyroid disruptors

#### Thyroid disruptors—general information

Although EDCs can affect the whole endocrine system, the thyroid gland and actually the whole hypothalamic-pituitary-thyroid axis seems to be the main target [24]. Numerous EDCs have been already identified as thyroid disruptors and mechanisms of their harmful effects have—at least partially—been known, comprising effects at the level of thyroid hormone synthesis, release, transport, metabolism, clearance, and hormone interactions with its receptor in target tissues [9, 24–27].

Regarding the direct action of EDCs on the thyroid gland, major targets seem to be the sodium/iodide symporter (Na^+^/I^−^ symporter, NIS), responsible for the active transport of iodine to the thyroid, and thyroperoxidase (TPO), the enzyme indispensable for thyroid hormone synthesis, but EDCs can disturb each step of hormonogenesis [24, 25].

Many EDCs affecting the thyroid gland are documented to act at the level of NIS, which mediates the uptake of iodide into the thyroid follicular cell. Numerous environmental chemicals have been identified for NIS inhibition activity in in vitro studies [28–30]. Because they compete with iodine, some of them are worth mentioning in this review and, therefore, they are shortly discussed below. Main NIS inhibitors are perchlorate (ClO_4_^−^), thiocyanate (SCN^−^), and nitrate (NO_3_^−^), with the first one being the most potent [31]. These three NIS inhibitors can be present in drinking water, however not exceeding usually safe concentrations, as it has recently been confirmed for example in different districts in Antalya (Turkey) [32].

Perchlorate comes from natural sources and can be found in food and drinking water, but it is also made in large quantities in the production of explosives. It is documented that exposure to perchlorate may disturb thyroid hormone synthesis at any age, especially in individuals with underlying thyroid disease or with iodine insufficiency [33]. Strong exposure of pregnant patients to perchlorate may result in a worse neurocognitive and behavioral development outcome in progeny independent of maternal thyroid hormone levels [34]. Perchlorate has a higher affinity to the NIS than iodine [35]. Because it is so potent competitive inhibitor of NIS, it is used in pharmacological doses to treat some types of thyrotoxicosis resulting from excess iodine [33]. Whereas perchlorate strongly inhibits iodine uptake at the level of NIS, it is not transported at all by NIS [31], and, therefore, it does not—in opposite to thiocyanate and nitrate—affect other steps of thyroid hormone synthesis (it has been reported to have no effect on iodine organification) [35].

Thiocyanate is a degradation product of chemicals found in cigarette smoke, in vegetables (such as cassava), and, generally, in different types of food. Although thiocyanate is much less potent than perchlorate in NIS downregulation, it inhibits also TPO and modifies iodine efflux from the thyroid [36]. Humans are broadly exposed to thiocyanates, which however—at environmental levels—do not cause substantial decrease in thyroid hormone concentrations, but may result in a well-known phenomenon of goiter formation. It has been recently documented that exposure to thiocyanate in excess is associated with the increased prevalence of goiter in area with iodine sufficiency (a rural area of northeast India) [37]. Importantly, thiocyanates may increase the risk of thyroid disruption in populations co-exposed to other EDCs.

Recently published results indicate that adolescents constitute a particularly sensitive population to perchlorate and thiocyanate regarding their blocking effect on NIS [38].

Nitrates, although produced naturally, have carcinogenic properties due to the endogenous formation of N-nitroso compounds. They can act as EDCs as they are NIS inhibitors, inhibit TPO and thyroglobulin (Tg, thyroid glycoprotein required for thyroid hormone synthesis), and generate reactive oxygen species (ROS); consumption of water contaminated with nitrates may affect thyroid function in humans [39].

Regarding the effects of bisphenol A on the thyroid gland, numerous studies (experimental, clinical observational, and epidemiological) were performed and related papers have been published; they were recently summarized in bibliometric analysis [40]. Generally, it can be stated that bisphenol A disrupts or antagonizes thyroid function [40], which is in agreement with the inverse association between the level of this EDC and thyroid hormone concentrations [41, 42]. Interestingly, a positive association was found between bisphenol A and occurrence of thyroid nodules in women with positive thyroid antibodies [43], which could also result from diminished thyroid function. Regarding neonates and children, bisphenol A seems to exert unfavorable effects on thyroid function in these sensitive groups; however, the results are inconclusive [44]. Although mechanisms of bisphenol A action are probably diverse, it should be underlined with respect to the topic of the present review that this EDC increases the generation of hydrogen peroxide (H_2_O_2_), being one of the basal forms of ROS, in vitro and in vivo models (via increasing DUOX2 [the enzyme responsible for H_2_O_2_ generation in the thyroid] mRNA level) and decreases iodine uptake (reduces NIS mRNA level) and TPO activity [45].

**Fig. 1:**
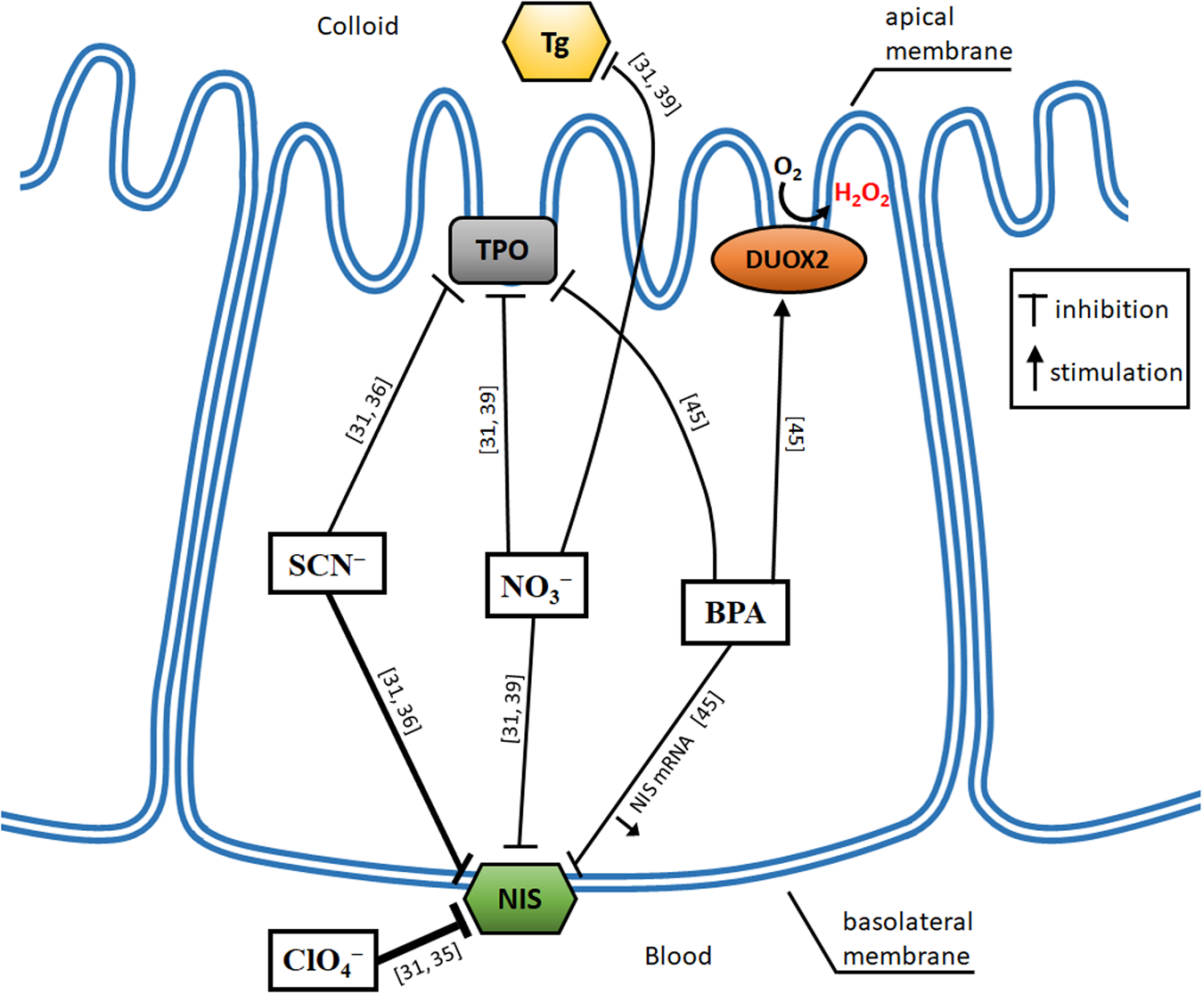
Mechanisms of disrupting effects of four confirmed EDCs (ClO_4_^−^, perchlorate; SCN^−^, thiocyanate; NO_3_^−^, nitrate; BPA, bisphenol A) on thyroid hormone synthesis. NIS sodium/iodide symporter; TPO thyroperoxidase; Tg thyroglobulin; DUOX2 dual oxidase 2. Thickness of graphic symbol indicating inhibition (┬) illustrates the potency of action of a given chemical. Numbers of references are written in square brackets

Mechanisms of disrupting effects of above four confirmed EDCs on thyroid hormone synthesis are illustrated in Fig. 1.

It should be mentioned that classic antithyroid medications used to treat hyperthyroidism, such as propylthiouracil (PTU) and methimazole, inhibit TPO, which results in decreased thyroid hormone synthesis; thus, these medications act as thyroid disruptors. It has been shown recently that amitrole, present in the environment triazole herbicide, reduced—similarly to methimazole—serum thyroxine concentrations in a dose-dependent manner [46].

Other thyroid disruptors broadly described in the literature are polychlorinated biphenyls, polybrominated diphenyl ethers, pesticides, phthalates, and perfluoroalkyl substances [9, 24, 25].

It is to be recalled that still increasing prevalence of autoimmune thyroid diseases is attributed, at least to a certain extent, to environmental EDCs, as they are observed more commonly in individuals living in polluted areas near petrochemical plants or aluminum foundries, in petrochemical workers, in areas contaminated with organochlorine pesticides, polychlorinated biphenyls, etc. [47].

There is concern about exposure to thyroid disruptors during pregnancy. They can interfere with thyroid hormone action resulting in potential unfavorable effects on fetal neurodevelopment and, thereafter, on the outcome in newborns, infants, and children [48, 49]. This issue has been elegantly summarized in a recently published review paper; detrimental risks and long-term consequences after in utero exposure to external factors either recognized as thyroid disruptors (perchlorate, phthalates, bisphenol A) or those with potential thyroid disruption activity (parabens, pesticides, and persistent organic pollutants) have been described in several cohort studies [50].

There is a need to more effectively identify thyroid disruptors, therefore still new strategies have been developed [51]. However, we should realize that to identify a given agent as a thyroid disruptor (and also any EDC) meets numerous difficulties, for example, exposure to usually only one (or just a few chosen) potential EDC under experimental conditions, and even our still limited knowledge regarding physiological processes in the thyroid resulting in diagnostic doubts which thyroid hormone concentration is normal or abnormal, etc. [52].

#### Iodine as a thyroid disruptor

Iodine and iodine-containing compounds such as povidone-iodine were initially considered as possible endocrine disruptors [53]. However, it has not been confirmed that estrogen-, androgen-, and steroidogenic-related activity is attributable to iodine (therefore not all criteria of EATS pathway are fulfilled) and no toxicologically significant adversity was observed in the available data set [54]. Regarding thyroid pathway, potential endocrine disruption of thyroid function caused by iodine was not considered as this micronutrient is indispensable for thyroid hormone synthesis and because this issue is “possibly beyond the scope of the Commission Delegated Regulation (EU) 2017/2100” [54]. It is worth mentioning that the above standpoint is based on literature review of 23 full-text documents (from 381 records retrieved from bibliographic databases) in which only four articles provided relevant information on the potential endocrine disruptive properties of iodine [55]. Nevertheless, in the context of the potential endocrine-disrupting properties of iodine new studies should be carried out.

Interestingly, the iodinated contrast media (ICM), which have been detected at high concentrations in surface water systems and also in fish organs, have recently been shown to interact with nuclear receptors that play key roles in endocrine regulation, including androgen and estrogen receptors; it should be mentioned that one of ICM, i.e., iohexol, revealed stronger binding than natural thyroid hormones [56]. Thus, the potential of ICM present in the environment to act as EDCs should be considered.

#### Iodine deficiency enhances damaging effects of thyroid disruptors

The data from epidemiological and experimental studies strongly suggest that iodine deficiency enhances damaging effects of thyroid disruptors, whereas iodine sufficiency reveals a kind of protection against them. Only some examples from the literature regarding this issue are mentioned below.

Association between exposure to perchlorate and blood serum thyrotropin (TSH—the pituitary hormone being the main stimulatory factor for thyroid hormone synthesis; its blood concentration is usually inversely associated with blood concentrations of thyroid hormones) was stronger in women with iodine deficiency (measured as urine iodine concentration (UIC)) than in iodine sufficient women, and also stronger in smoking women, the last association being probably due to the action of thiocyanates (present in cigarette smoke) that also inhibit iodine uptake [57, 58].

More pronounced effect regarding mild thyroid dysfunction caused by occupational exposure to ethylenebisdithiocarbamates, a fungicide widely used in agriculture, was observed in workers residing in an area with mild or moderate iodine deficiency comparing to workers living in an area covered by a long-lasting iodine prophylaxis program [59].

Regarding the opposite condition, i.e., optimal iodine supply, it has been presented in a recently published review that nutritional interventions, including iodine supplementation, are able to ameliorate the effect of endocrine disruptors on human reproductive health [60].

In addition, exposure to EDCs may enhance the consequences of iodine deficiency. In a population study on iodine deficiency and thyroid disruptors performed in children (under the age of 7 years) in Haiti, a positive association was found between exposure to perchlorate or thiocyanate and median urinary iodine levels. Such results suggest that competitive inhibition of iodine uptake in the thyroid caused by these two EDCs results in increased iodine excretion in the urine [61]. Thus, in populations exposed to excessive amounts of EDCs (being NIS inhibitors), not only the real iodine uptake in the thyroid can be lower with all its consequences, but also urinary iodine concentration may falsely suggest adequate iodine supply.

## The role of iodine in living organisms

Iodine is a non-metallic micronutrient essential for the synthesis of thyroid hormones, i.e., thyroxine (3,5,3′,5′-tetraiodo-L-thyronine, T_4_) and triiodothyronine (3,5,3′-triiodo-L-thyronine, T_3_) and this is the main role of iodine in humans [62, 63] (see also the subsection “The role of oxidative stress in thyroid hormone synthesis—general information”). Thyroid hormones play a crucial role in regulating metabolic processes, growth, and development via various mechanisms [64].

However, the role of iodine in living organisms extends beyond that of thyroid hormone synthesis [63, 65]. Other physiological effects of iodine are as follows. Iodine possesses a significant activity as a scavenger of ROS; therefore, it is an effective antioxidant in living organisms [65, 66]. As iodide is oxidized to hypoiodite anion (IO^−^) it reveals strong bactericidal, antiviral, and antifungal effects [67–72]. It is documented to be an apoptotic, antiproliferative, and differentiation factor in various tissues [65, 66]. Of great importance are findings showing that iodine is able to exert antineoplastic effects, the issue which has been recently reviewed [73]. Protective effects of iodine against process of carcinogenesis have been demonstrated regarding breast cancer, stomach cancer [74, 75], as well as different human cancer-derived cell lines [63, 76]. Iodine is thought to take part in innate immune defense, among others in the gastrointestinal tract [63, 65, 73]. All the above effects at molecular levels driven by iodine may—under favorable conditions—contribute to defense against numerous disorders, such as infection, inflammation, cancer initiation and progression, etc.

Living organisms are unable to produce iodine, which must be delivered with food as iodide (I^−^), iodate (IO_3_^−^), or organically bound iodine, and then absorbed in the intestine in the form of inorganic iodide [62]. The total body iodine content in humans was estimated to be approximately 12–25 mg, of which 5–15 mg is stored in the thyroid [77]. An important role in iodine homeostasis is played by NIS [78]. This protein is responsible for the active transport of I^−^ driven by the electrochemical gradient of sodium ion (Na^+^) into the thyroid gland at the level of the basolateral membrane [79].

Iodine deficiency may have many clinical implications and leads to diseases resulting from inadequate thyroid hormone synthesis, termed iodine deficiency disorders (IDD) [68]. The classic consequence of iodine insufficiency in all age groups is goiter, resulting from physiological adaptation to chronic iodine deficiency [68, 69]. Severe iodine deficiency may result in hypothyroidism [67], and when occurs during pregnancy may cause stillbirths, miscarriages, and cretinism associated with other congenital abnormalities [68].

The main indicators of iodine deficiency in a given area include the decreased UIC, the increased prevalence of goiter, and the increased blood Tg concentration [80].

## Iodine prophylaxis

### Models of iodine prophylaxis and iodine compounds

Universal salt iodization is widely recognized as the most cost-effective method to reduce iodine deficiency [81, 82]. Currently, most countries in the world have legislation for mandatory salt iodization and some others—for voluntary iodization. As a result, over one hundred countries in the world have sufficient iodine supply [83]. At the same time it should be mentioned that 13 countries have excessive iodine intake [83].

Programs of salt iodization are based on the use of either potassium iodide (KI) or potassium iodate (KIO_3_), with the latter—due to its higher stability and less soluble form—being the most commonly used iodine compound for this process, especially in moist tropical locations [82, 84]. Iodate is a strong oxidant and the results of some experimental studies suggest that iodate intake may cause adverse effects. Regarding the safety of KIO_3_ to humans, no danger has been confirmed in numerous studies; therefore, Food and Drug Administration has given this compound GRAS status, which means “generally recognized as safe” [85]. In addition, it has been documented that during cooking above 96% of iodate present in salt added to food is converted into either iodide (almost 87%) or molecular iodine (almost 10%) [86].

Programs of salt iodization with the use of either KIO_3_ or KI is effective in most countries [83], with the example of Poland [87].

Regarding iodine given in tablets, KI is practically the only compound used.

### Recommended iodine doses

Recommended iodine doses depend on age ranges and distinctive physiological states, as recommended by National Institutes of Health, WHO, and the American Thyroid Association [88–90], with very small differences between the above guidelines. For adult nonpregnant population, the daily dose of 150 µg is recommended [88, 89]. Regarding pregnant and breastfeeding patients, and also women planning pregnancy, iodine supply of 250 µg/d is recommended [89, 90]. Children require generally lower iodine doses than adults [89].

The above iodine requirements are covered in most countries by universal salt iodization. Only women planning pregnancy, pregnant, and breastfeeding patients should take additionally approximately 150 µg/d of iodine (as KI) in tablets [90].

The recommended biomarker of population iodine status is median UIC (mUIC) [91]. More than 90% of dietary iodine is excreted within 24–48 h, so excretion of iodine in the urine is a good marker of recent iodine intake. Adequate iodine intake is defined as mUIC in the range of 100–299 μg/L in school children and non-pregnant adults, and adequate and more than adequate—in the range of 150–249 μg/L and of 250–499 μg/L, respectively, in pregnant women [83, 89, 91].

## Iodine excess

### Sources of iodine excess

Although the relatively small risk of iodine excess is far outweighed by the substantial risks of iodine deficiency, the former should not be neglected.

Accordingly to mentioned above appropriate normal mIUC, the upper threshold for iodine excess is mUIC ≥300 μg/L in school children and ≥500 μg/L in pregnant women [89]. In areas with previously occurring iodine deficiency these thresholds are lower, i.e., mUIC ≥200 μg/L [91].

Iodine supplementation must be carefully monitored to ensure adequate iodine intake while avoiding iodine excess. Exposure to iodine excess may have numerous consequences [22]. The increased exposure to iodine can cause subclinical or overt thyroid dysfunction [22], also of autoimmune origin [92, 93], especially in individuals with preexisting thyroid disease, in elderly, fetuses, and neonates. As the proportion of papillary thyroid cancer (PTC) to follicular thyroid cancer increased substantially after the iodine prophylaxis had been introduced, it is hypothesized that excess iodine may also increase the risk of PTC [94].

Sources of iodine excess are precisely presented in some review papers [20, 91, 95]. From a practical point of view, they can be divided into three groups and the significance of this “classification” relies on that to what extent the exposure to iodine excess can be avoided.

The first group of sources relates to exposure to iodine excess from natural environment or from universal iodine use in some industrial procedures; in this case, all individuals (or majority) living in a given area are exposed to iodine excess. One of the most important factors of excessive iodine exposure is associated with regional nutritional habits. This is observed mainly in coastal areas in Asian countries, such as Japan or Korea, characterized by the common consumption of food rich in iodine seaweed [96]. Drinking water may be also a potential source of excess iodine, which was documented for example in China [97] or in some areas in Europe [98]. Another source constitutes iodine-containing water–purification tablets [99]. The important potential source of iodine excess is iodized salt in countries in which legislation stipulates salt iodine fortification above the recommended level or if salt is overiodized at production [91]. It should be stressed, however, that salt iodization at recommended level is not associated with exposure to iodine excess.

The second group comprises iodine supplements, mineral waters with known and recommended iodine concentration, milk and dairy products, which can constitute sources of iodine excess only if some individuals do not follow recommendations. Whereas rules regarding iodine supplementation during pregnancy and lactation are formulated, overtreatment with iodine-containing tablets in these groups of patients may put consumers at risk [91]. Bottled mineral waters with known iodine level, which are recommended to improve the effectiveness of iodine prophylaxis (especially in countries trying to adjust to optimal salt consumption), should contain iodine in the concentration of 100–200 μg/L. Thus, drinking for example 2 liters of such a water plus normal diet would overload the organism with iodine dose above estimated threshold. A separate issue is that certain types of mineral waters, available mainly in resorts, contain excessive amount of iodine. For example, it has been shown recently in the Polish study that iodine at the level of more than 500 µg/L and even almost 3000 µg/L is present in samples of such water available in resorts with a long tradition in the field of spa medicine [100]. It is obvious that drinking only one glass of such a water daily can be associated with exceeding the safe iodine threshold by several times. Milk and dairy products can constitute important sources of iodine with milk iodine concentrations ranging from several dozen to over 500 μg/L in industrialized countries [101]. Therefore, with high milk and/or dairy consumption some individuals may be at risk of iodine excess. It should be stressed that the exposure to iodine excess in case of the second group of iodine sources can be completely avoided if individuals adhere to the broadly known rules.

The third group of iodine-rich sources comprises chemicals that are used as either medications or in diagnostics. One of the most common is amiodarone, anti-arrhythmic drug, containing 75 mg of iodine in one 200 mg tablet [102]. Thus, even low dose oral therapy can elevate daily iodine intake by several hundred times comparing to recommended daily dose [102]. Also, other characteristics of this medication make it potentially dangerous in certain susceptible individuals causing thyroid dysfunction and/or thyroiditis [20, 103]. It is confirmed that amiodarone-induced hyperthyroidism is more common in iodine-deficient areas, whereas amiodarone-induced hypothyroidism is more common in iodine-sufficient areas, with the former being much more problematic regarding clinical course and management [20, 103]. Thus, adequate iodine supply prevents more serious consequences of iodine excess (see also the subsection “Iodine deficiency enhances damaging effects of thyroid disruptors”). The next source of iodine excess is ICM, of which a single dose can contain over 10 mg of bioavailable free iodine and up to 60 g of bound iodine (being several thousand times higher than the recommended daily iodine dose) [20, 104]. Iodine level in the body after the contrast administration will remain elevated for a time longer than 1 month [105]. Transdermal antiseptic cleaners containing povidone-iodine, which are frequently used in hospitals for hand washing or wound care, may constitute another source of iodine excess. Therefore, their long-time use may induce thyroid disorders [20]. Regarding the third group of iodine-rich sources, the consequences of exposure to iodine excess can be avoided in a substantial number of individuals by taking a more careful and well-thought-out decision to use these chemicals either in the treatment or in diagnostics.

Up to the levels of 1100 µg/d iodine is usually well tolerated in most people, except in certain susceptible individuals such as those with pre-existing thyroid disease, the elderly, fetuses, and neonates—these groups may have an increased risk of thyroid dysfunction [20].

Regarding exposure to iodine excess during pregnancy, no specific studies have been published so far. However, it should be taken into account that damaging effects of excessive iodine are more probable if fetal or neonatal thyroid is exposed to, as it does not possess the completely developed mechanism to escape from the Wolff–Chaikoff effect [20] (see also the subsection “Adaptation to iodine excess”).

### Adaptation to iodine excess

The thyroid gland has developed protective mechanisms to adapt to excess iodine.

The first study regarding this issue was conducted in 1944 and it revealed that in vitro exposure of sheep thyroid slices to large amounts of inorganic iodide resulted in inhibition of thyroid hormone formation [106].

The next milestone was the in vivo study performed by Wolff and Chaikoff who documented reduction in thyroid hormone synthesis in rats injected with high amounts of iodide, the phenomenon occurring when plasma iodide level achieved a critical threshold; the block of thyroid hormone synthesis lasted approximately 24 h [107]. This effect is known as the acute Wolff–Chaikoff effect [107]. The acute Wolff–Chaikoff effect may be partially explained by the generation of some inhibitory substances, such as intrathyroidal iodolactones, iodoaldehydes, or iodolipids, on thyroid peroxidase activity [35]. Importantly, while the organic binding of iodine was blocked, the thyroid gland was still able to concentrate iodine, which suggests that the normal thyroid possesses a mechanism for concentrating iodine that does not depend upon its conversion to thyroid hormones [107]. It has been generally accepted that the Wolff–Chaikoff effect is caused by iodine concentration of at least of 10^−3^ M [84, 95, 108].

The third step was to demonstrate that described above inhibitory effect of excess iodide was transient, lasting from 24–48 h; after this time interval of the inhibitory effect, an adaptation or escape takes place, and organification and thyroid hormone biosynthesis return to a normal state [109]. The escape phenomenon has not been completely understood; however, the results from experimental studies suggest that it is caused by a downregulation of NIS, resulting in decreased iodide transport into the thyroid. This would then decrease the intrathyroidal iodine concentration below a critical threshold and restore normal iodine organification and, in consequence, normal thyroid hormone biosynthesis [110].

Whereas mechanisms of both phenomenon, i.e., the Wolff–Chaikoff effect and escape phenomenon from this effect, are definitely complex, they also include elements of oxidative stress. Regarding the first phenomenon, results of the study performed more than 30 years ago suggest that the Wolff–Chaikoff effect is caused by inhibition of H_2_O_2_ generation [111]. Namely, in dog thyroid slices iodide strongly inhibited H_2_O_2_ generation stimulated by TSH and by carbamylcholine and it also inhibited the action of intracellular signals on the H_2_O_2_ generating system [111]. In the next studies, it has been confirmed that high concentrations of iodide, probably in the form of iodolipids, inhibit H_2_O_2_ generation [112, 113]. Iodolipids have been found to inhibit DUOX (generating H_2_O_2_) and adenylyl cyclase, suggesting that they are involved in regulatory actions of I^−^ in the thyroid via H_2_O_2_ [114]. Regarding the potential role of oxidative processes in the escape phenomenon, it is suggested that the generation of ROS [115–117] is responsible for NIS downregulation resulting from exposure to iodine excess [110] (see also the subsections “In vitro studies” and “In vivo studies”).

**Fig. 2:**
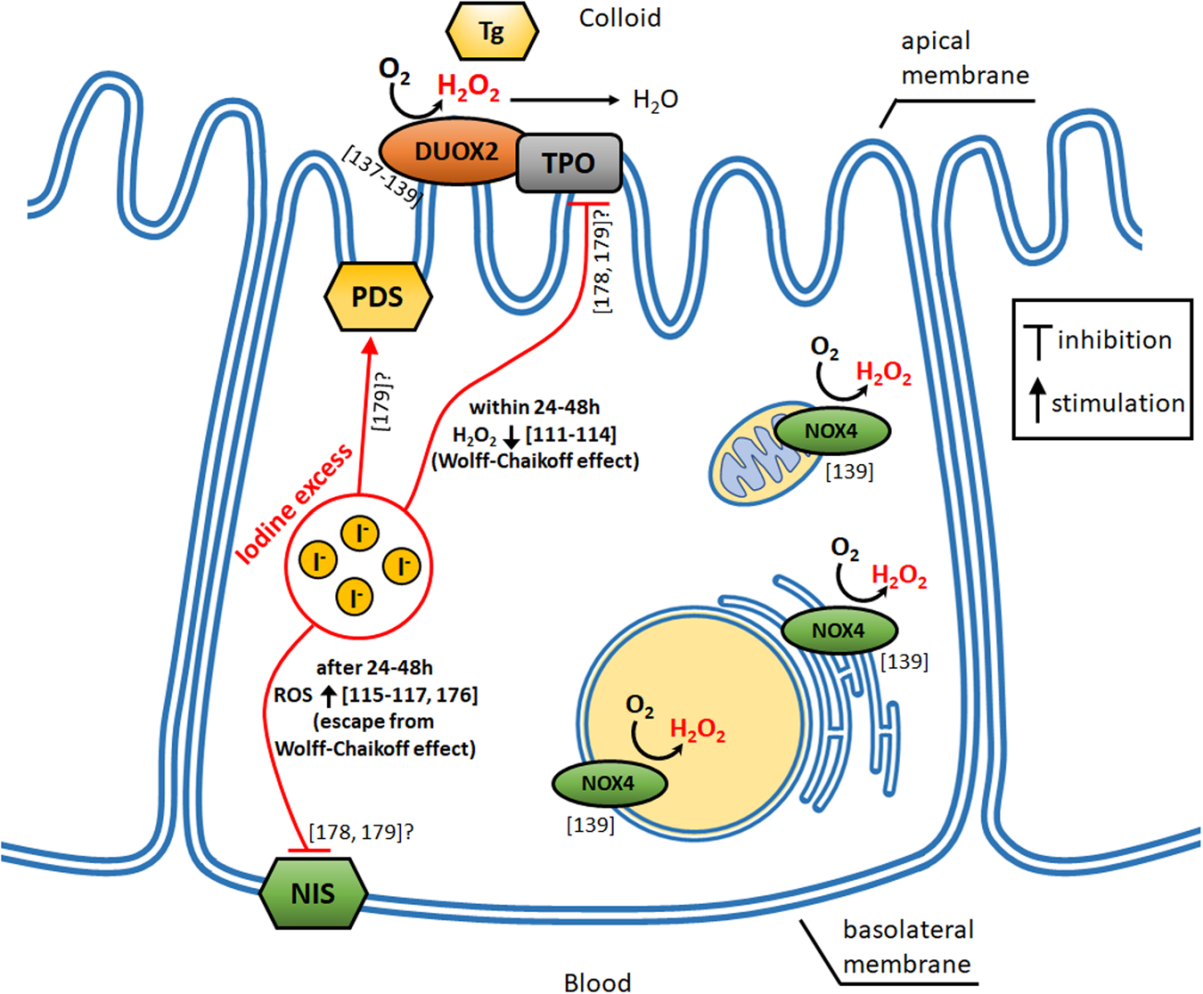
Mechanisms of disrupting effects of iodide (I^−^) on thyroid hormone synthesis. NIS sodium/iodide symporter; TPO thyroperoxidase; Tg thyroglobulin; PDS pendrin; DUOX2 dual oxidase 2; NOX4 NADPH oxidase 4. The question mark (?) designates undefined mechanisms of action. Numbers of references are written in square brackets

Mechanisms (taking also into account components of oxidative stress) of disrupting effects of iodine excess on thyroid hormone synthesis are illustrated in Fig. 2.

The mechanism of the Wolff–Chaikoff effect can practically be used in the event of uncommon emergencies like nuclear or radiological incidents. If individuals are exposed to radioiodine they are recommended to take a high dose of stable iodine (up to 100 mg depending on the age) to avoid radioiodine uptake by the thyroid [118]. Such an action was carried out in Poland following the Chernobyl reactor accident [119, 120]. Whereas repeated doses of stable (non-radioactive) iodine are accepted in most individuals of populations exposed to radioiodine for a long time, neonates, pregnant, and breastfeeding women should not receive repeated iodine doses due to the risk of adverse effects [118].

Because the Wolff–Chaikoff effect caused by excess iodine is only transient (24–48 h), experimental pharmacokinetic models have been developed to look for longer protection against chronic radioiodine exposure. With the use of such a model, recently published results have revealed high effectiveness of non-radioactive iodine (100 mg) in case of acute radioiodine exposure, but in case of prolonged exposures higher protection was revealed by perchlorate in the recommended dose of 1000 mg [121]. In the next study, the same authors applied pharmacokinetic models mimicking two different populations regarding iodine supply, i.e., Caucasians and Japanese, the latter with much higher nutritional iodine intake [122]. In Caucasians, iodine and perchlorate were effective to the same extent in case of acute radioiodine exposure; however, in case of continuous exposure, a stronger protection was revealed by perchlorate. In turn, perchlorate showed superiority in case of both, acute and long-lasting exposure to radioiodine, in Japanese [122]. The authors conclude that perchlorate reveals superiority over stable iodine in populations with very high iodine supply independent of radioiodine exposure is acute or prolonged, and also in areas with normal iodine supply when exposure to radioiodine is long-lasting [122]. Although these considerations are at this moment rather theoretical, perchlorate administration is recommended in the individuals exposed to radioiodine who reveal hypersensitivity to iodine [118].

## The role of oxidative stress in thyroid physiology

### The role of oxidative stress in thyroid hormone synthesis—general information

ROS play an essential role in different physiological processes; consequently, ROS underproduction can contribute or even cause some abnormalities resulting in certain diseases [123]. Among different ROS a prominent position is attributed to H_2_O_2_, being the diffusible second messenger that serves as signaling molecule in redox processes [123].

Whereas oxidative reactions occur in almost all tissues and organs, the thyroid gland appears to be an organ of “oxidative nature”, in which ROS plays a particular role in thyroid physiology [124]. The most important elements indispensable for thyroid hormone biosynthesis are iodide, TPO, H_2_O_2_, and Tg [125], among which iodine is the only exogenous element. Four or three iodine atoms are present in chemical structures of thyroid hormones, i.e., 3,5,3’,5’- tetraiodo-L-thyronine (thyroxine; T_4_) or 3,5,3’- triiodo-L-thyronine (T_3_), respectively [125].

The synthesis of thyroid hormones requires the transport of iodine into thyroid cells. As mentioned earlier, NIS is responsible for this transport [78, 125, 126]. NIS is a transmembrane protein located on the basolateral membrane of thyroid follicular cells, which pumps two cations of Na^+^ and one I^–^ anion from the bloodstream into cells [78]. In the next step, iodine anion is transported across the apical membrane into the colloid of the follicular lumen [125]. In the colloid, the enzyme TPO catalyzes the oxidation of I^–^ and iodination of the tyrosyl residues of Tg molecules to generate iodotyrosines, i.e., monoiodotyrosine (MIT) and diiodotyrosine (DIT). One MIT and one DIT form T_3_ or two molecules of DIT create T_4_. Then iodinated Tg is absorbed again by the action of TSH into thyroid cells, where it is digested by proteases to release thyroid hormones into circulation [125, 127].

H_2_O_2_ acts as an electron acceptor at each step of thyroid hormone synthesis, i.e., oxidation from iodide to iodine by the enzyme TPO, organification of iodine, and at coupling reaction of iodotyrosines. H_2_O_2_ availability is the rate-limiting step in thyroid hormone biosynthesis (see also the subsection “The role of H_2_O_2_ in thyroid physiology”), although this molecule is produced in large excess, when compared with the amount of iodide incorporated into proteins [128]. Not only H_2_O_2_ but also other reactive species are formed in the thyroid gland, e.g., nitric oxide (NO^●^) [129]. Moreover, some elements of thyroid hormone synthesis pathway are partially confirmed to exist as ROS, such as tyrosine free radical (Tyr^●^), diiodotyrosyl residue radical (DIT^●^), diiodotyrosyl residue radical in Tg (Tg-DIT^●^), iodine radical (I^●^), iodinium ion (I^+^), hypoiodous acid intermediate [IO^–^], and ascorbate radical (Asc^●^) [124]. As mentioned above, oxidative processes participate in the Wolff–Chaikoff effect and in the escape phenomenon (see also the subsection “Adaptation to iodine excess”).

For this reason, the thyroid gland has developed an antioxidative defense system that is composed of antioxidative enzymes and free radical scavengers [130], comprising among others superoxide dismutase (SOD), glutathione peroxidase (GSH-Px), and catalase (CAT), vitamins C and E, α- and γ-tocopherols or coenzyme Q [124, 130]. Among antioxidants whose presence has been documented in the thyroid, peroxiredoxins (Prxs) are of a special significance, as they are involved in the process of H_2_O_2_ elimination, when this ROS is formed in response to TSH, and they protect thyroid cells from H_2_O_2_-induced apoptosis [130, 131].

It has recently been shown that mutations of genes encoding for nicotinamide nucleotide transhydrogenase, the enzyme playing an important role in the regulation of redox homeostasis, result in clinical phenotype of hypothyroidism [132]. Such results strongly suggest a crucial role of oxidative processes in thyroid physiology. In addition, iodide efflux at the apical membrane of FRTL-5 thyrocytes was found to be mediated by ROS production induced by iodine excess [133].

Under physiological conditions, there is a balance between the production of ROS, which are required for thyroid hormone synthesis, and the action of the endogenous antioxidative system in the thyroid. Any imbalance between these processes may result in increased oxidative damage to macromolecules and, in consequence, certain thyroid abnormalities [124].

The statement that the thyroid gland is an organ of “oxidative nature” is also justified by the observations that the thyroid versus other different tissues is less sensitive to damaging effects of prooxidative agents such as KIO_3_ [134] or Fenton reaction substrates [135, 136].

### The role of H_2_O_2_ in thyroid physiology

Hydrogen peroxide (H_2_O_2_) is a form of ROS. All cells produce continuously low or moderate amounts of H_2_O_2_ under physiological conditions. This ROS is generated mainly by the intracellular family of NADPH oxidases (NOX). Hydrogen peroxide, which is an essential factor for thyroid hormone biosynthesis, is produced in the thyroid gland by two isoform enzymes, i.e., dual oxidase 1 (DUOX1) and 2 (DUOX2), belonging to NOX family, with the most convincing experimental evidence found for DUOX2 [137–139]. Both of them are expressed in the apical plasma membrane of thyroid follicular cells (thyrocytes) [137, 138]. The next enzyme which also plays an important role in H_2_O_2_ synthesis in the thyroid is NOX4, acting intracellulary [139]. Hydrogen peroxide acts as an electron acceptor at each step of thyroid hormone synthesis, namely iodide oxidation and, next, its organification, as well as coupling reaction of iodotyrosines [124, 140]. It is essential for TPO activity.

It is supposed that H_2_O_2_ produced by NOX4 does not participate in thyroid hormone synthesis and, therefore, it may contribute to some pathological processes to a greater extent than that one produced by DUOX. NOX4 produces H_2_O_2_ in the perinuclear region within thyroid cell, thus, when being in excess it can induce DNA damage, and also in such intracellular compartments as mitochondria and endoplasmic reticulum [139] (see also Fig. 2).

TPO is a heme-dependent protein, thus it contains iron atoms in its structure. Hydrogen peroxide participates in autocatalytic covalent heme binding to the apoprotein of TPO molecule, thereby stabilizing the activity of the enzyme [141]. As it has been mentioned above, H_2_O_2_ availability is the rate-limiting step in thyroid hormone biosynthesis. Because of the relatively high Michaelis–Menten constant of TPO for H_2_O_2_, relatively high concentrations of H_2_O_2_, as a substrate, are required to activate the enzyme [128, 142]. Large quantities and membrane permeable nature of H_2_O_2_ can lead to its diffusion from the luminal side of the apical membrane back to the cell.

TSH, the main factor stimulating secretion and growth processes in the thyroid, is obviously involved in the synthesis of H_2_O_2_ in that gland; it has been shown that TSH stimulates DUOX activity in porcine thyroid cells [143] and that it stimulates *DUOX2* promoter (DNA sequence controlling transcription initiation) activity [144].

### The role of H_2_O_2_ in pathological processes in the thyroid gland

Because iron is present in TPO and H_2_O_2_ is required for the activity of TPO, under certain conditions the thyroid gland may be exposed to excessive amounts of either ferrous ion (Fe^2+^) or H_2_O_2_, or both. At such circumstances, favorable conditions are created for additional Fenton reaction (Fe^2+^ + H_2_O_2_ → Fe^3+^ + ^●^OH + OH^–^), and, consequently, for the enhanced oxidative damage to macromolecules. The major meaning of that fact is that increased production of H_2_O_2_, with subsequently increased formation of free radicals, takes place in any conditions accompanied by the increased blood TSH concentration. Because TSH stimulation results in goiter formation and, under certain conditions, in thyroid cancer initiation, both these pathologies occur partially via the mechanism of oxidative stress. In agreement with this, oxidative stress creates the required conditions for thyroid cell proliferation [145, 146]. On the other hand, recently published results suggest that H_2_O_2_ in proper amounts is required to avoid goiter formation; namely, reduced affinity of TPO for H_2_O_2_ was associated with the formation of severe multinodular goiter in mice [147].

In agreement with H_2_O_2_ contribution to goiter formation is its contribution to thyroid cancer initiation. H_2_O_2_ in excess was found to induce DNA oxidation, mutagenesis, the activation of proto-oncogenes, or inactivation of tumor suppressors genes, all leading to proliferative effects and neoplastic events [148]. H_2_O_2_ and possibly other elements of oxidative stress may be involved also in the coexistence of congenital hypothyroidism and thyroid cancer [149].

It has recently been observed that H_2_O_2_ participates in the regulation of DNA repair mechanisms in normal thyroid cells, whereas this process was dysregulated in cancerous cells (a model of the PTC with RET/PTC rearrangement) [150].

It is worth mentioning that in numerous experimental studies Fenton reaction substrates, one of which is H_2_O_2_, are repeatedly confirmed to increase oxidative damage to macromolecules in different tissues (reviewed in, e.g., [151], also [135, 152–155]), the thyroid gland included [135, 156–159].

Not only H_2_O_2_ excess but also thyroidal H_2_O_2_ deficiency may have serious clinical consequences. It is known that genetically determined alterations in DUOX2 and its maturation factor (DUOXA2), which is a very rare phenomenon, result in congenital severe hypothyroidism [160].

### Oxidative properties of iodine

Oxidative properties of iodine are well documented. As mentioned above, I^–^ acts as an electron donor and it is oxidized by TPO in the process of thyroid hormone synthesis [125]. Moreover, these reducing properties make it an important free radical scavenger [65, 66] and, as it has been mentioned earlier, via oxidation to hypoiodite anion (IO^–^)—a potent oxidant with strong bactericidal activity [72].

Two main compounds of iodine used in iodine prophylaxis, i.e., KI and KIO_3_, have different pro- and antioxidative properties. KIO_3_ reveals oxidizing properties whereas KI is neutral. The differences between the oxidative properties of KI and KIO_3_ and their effects on oxidative damage to macromolecules in the thyroid gland were documented recently by us in experimental studies [158, 159, 161].

The redox potential of KIO_3_ is 1.195 V in acidic and 0.56 V in neutral environments, both values being higher than those of KI [162]. IO_3_^–^ should be reduced to I^–^ to be effectively used by the thyroid, and this process may consume many hydrogen ions and electrons [162, 163]. Interestingly, high concentrations of KIO_3_ may also weaken the antioxidant capabilities of tissues [162].

With respect to DNA damage, it has been shown that iodine oxidizes 2’-deoxy-8-oxoguanosine [164] and 8-oxo-7,8-dihydroguanine [165], both resulting from oxidative damage to guanine bases.

## Oxidative damage to macromolecules caused by iodine compounds

Recently many studies have been conducted regarding potential oxidative damage to macromolecules caused by main iodine compounds, i.e., KI and KIO_3_, used in worldwide strategies for prevention of iodine deficiency. Whereas KI seems to be completely safe, KIO_3_ is recommended preferentially in salt iodization programs due to its greater chemical stability. Iodate was tested for its potential toxicity, but this hypothesis has not been confirmed till now in humans. However, it should be taken into account that iodic acid (HIO_3_), from which iodate salts are formed, belongs to the group of oxohalogen acids comprising also chloric acid (HClO_3_) and bromic acid (HBrO_3_). Whereas KIO_3_ is characterized by the lowest redox potential among these three halogenate salts, potassium bromate (KBrO_3_), a known potential renal carcinogen [166] belonging to the group 2B (“possibly carcinogenic to humans”) according to IARC classification [167], has a similar chemical structure to KIO_3_ and for this reason, it cannot be excluded that iodate may also be potentially dangerous, at least in experimental conditions.

### In vitro studies

The physiological iodine concentration in rat and human thyroid was calculated to be approximately 9.0 mM [168–170]. Taking into consideration the similarity between porcine and human thyroid (volume, the process of thyroid hormone synthesis) [171], it may be estimated that the concentration of iodine in porcine thyroid—broadly used in in vitro studies—is similar. Due to the very high molecular mass of iodine, molecular masses of KI and KIO_3_ are of the same order of magnitude. Therefore, if these compounds are used in experimental models, effects of either the whole compounds or iodide ions (I^−^) (formed from KI or KIO_3_) can be measured.

We have evaluated in the in vitro studies the following parameters of oxidative damage to macromolecules: oxidative damage to mitochondrial DNA (mtDNA) and nuclear DNA (nDNA) measured as the level of 8-oxo-7,8-dihydro-2’-deoxyguanosine, and oxidative damage to membrane lipids (lipid peroxidation) measured as the concentration of malondialdehyde + 4-hydroxyalkenals (MDA + 4-HDA) [e.g., 134, 152, 158, 159, 161, 172].

In the in vitro study with the use of porcine thyroid tissue, we have shown that under basal conditions (without additional pro-oxidative abuse), both iodine compounds, i.e., KI and KIO_3_ (2.5–50 mM) do not affect oxidative damage to DNA (both nDNA and mtDNA); therefore, they can be considered as safe with respect to their potential oxidative damage to thyroid DNA [158]. It should be stressed that the lowest concentration of iodine used in this study, i.e., 2.5 mM, corresponds to the same order of magnitude of iodine concentrations causing Wolff–Chaikoff effect, i.e., 10^−3^ M (1 mM) [95]. When iodine compounds were used together with Fenton reaction substrates, KI—in all used concentrations (therefore also in this corresponding to the concentration causing Wolff–Chaikoff effect)—completely prevented experimentally induced oxidative damage to mtDNA, whereas KIO_3_ was preventive only in its highest used concentrations of ≥25 mM [158]. We concluded that without additional prooxidative abuse, both iodine compounds, i.e., KI and KIO_3_, seem to be safe in terms of their potential oxidative damage to DNA in the thyroid. The superiority of KI over KIO_3_ relies on its stronger protective effects against experimentally induced oxidative damage to mtDNA, which constitutes an argument for its preferential utility in iodine prophylaxis [158].

Regarding iodine effects on oxidative damage to membrane lipids the following results were obtained by us [134, 159, 161, 172]. Porcine thyroid homogenates were incubated in the presence of either KI (0.00005–500 mM) or KIO_3_ (0.00005–200 mM).

KI increased lipid peroxidation only when used in its highest concentrations of ≥50 mM, but used in the middle range of concentrations (5.0; 10; 25; 50 and 100 mM), comprising also those corresponding to physiological concentration of iodine, it reduced Fenton reaction-induced oxidative damage. In opposite, KIO_3_ did increase lipid peroxidation in as low concentrations as ≥2.5 mM, and the strongest damaging effect was observed at the KIO_3_ concentration of 10 mM, corresponding to physiological iodine concentration in the thyroid [134, 161, 172]. KIO_3_ not only increased oxidative damage to membrane lipids by itself but it also—at concentrations of 5–200 mM—enhanced Fenton reaction-induced lipid peroxidation with the strongest damaging effect observed again for the concentration of 10 mM [159]. We have concluded that KI, used in doses resulting in physiological iodine concentrations, may prevent oxidative damage to membrane lipids in this gland, and that KIO_3_ does not possess any direct beneficial effects on oxidative damage to membrane lipids in the thyroid.

The superiority of KI over KIO_3_ in our in vitro experiments relies on its more favorable effects on oxidative damage to thyroidal mtDNA and membrane lipids under basal and experimentally induced oxidative damage [134, 158, 159, 161, 172].

The increased oxidative stress caused by iodate can be diminished by antioxidants, of which indole substances play an important role. In our in vitro studies, melatonin (a well-known antioxidant synthesized mainly in the pineal gland) and indole-3-propionic acid (a molecule similar in structure to melatonin) reduced lipid peroxidation induced by KIO_3_, used at concentrations close to physiological, in porcine thyroid homogenates [134, 161, 172]. Their cumulative effect was even stronger than when these two antioxidants were applied separately [172]. Other tissues were also sensitive to protective action of melatonin, but these effects were not as strong as in thyroid tissue [134].

Compared to the thyroid gland, other tissues contain only traces of iodine and the ratio of the iodine concentration in some organs (e.g., kidney, liver, skin, muscle) to that in the thyroid gland is approximately 1:100,000 [173]. Despite this, damaging effects of KIO_3_ (used again in concentrations of approximately 10–15 mM) were observed also in other tissues [134, 162]. It should be stressed that lipid peroxidation resulting from exposure to KIO_3_ was significantly lower in the thyroid gland than in other tissues [134], which indicates that there is a kind of adaptation of this gland to maintain large concentrations of iodine (this observation has been also mentioned in the subsection “The role of oxidative stress in thyroid hormone synthesis—general information”). Also in the kidney lower lipid peroxidation was found comparing to other tissues [134], and the explanation can be as follows. As mentioned above, KBrO_3_, as a known potential renal carcinogen, has a similar structure and chemical properties to KIO_3_, and therefore kidney tissue is, at least hypothetically, more resistant to damaging oxidative effects caused by not only bromate but also iodate [134].

In an experimental study with the use of human thyroid follicles isolated from normal paranodular tissue, it was observed that a high iodide dose (10^−3^ M) caused marked inhibition of radioiodine accumulation and organification process, as well as it induced necrosis of thyrocytes and ultrastructural lesions such as apical blebbing, cytoplasmic fragments desquamation, endoplasmic reticulum vesiculation, and accumulation of lipofuscin in secondary lysosomes [174]. This direct acute toxic effect of iodide is probably mediated by ROS, although not examined in that study.

When immortalized thyroid cell line (TAD-2), primary cultures of human thyroid cells and cells of nonthyroid origin were incubated in the presence of iodine in different concentrations, iodine dose-dependent cytotoxicity in both TAD-2 and primary thyroid cells were observed, with no effect on cells of nonthyroid origin [175]. Treatment with iodide excess resulted in morphological changes, plasma membrane phosphatidylserine exposure, and DNA fragmentation, features typical for apoptosis. In addition, KI treatment of TAD-2 cells increased ROS production and it dramatically increased the level of thiobarbituric acid reactive substances, being the index of lipid peroxidation [175]. These results indicate that excess iodide induces apoptosis in thyroid cells through a mechanism involving the generation of free radicals [175].

On the basis of experimental in vitro studies, it has been strongly suggested that the inhibitory effect of iodine excess on NIS expression involves the generation of ROS. With the use of PCCl3 thyroid cells, it has been documented that iodide, used in concentrations corresponding to those causing Wolff–Chaikoff effect (10^−3^ M to 10^−6^ M), increased ROS production in thyroid follicular cells; expectedly, the use of ROS scavengers blocked the effect of iodide excess on Akt phosphorylation (as an element of the PI3K/Akt signaling pathway documented, among others, to downregulate NIS) [116]. Next, the same authors have confirmed that I^−^ excess transcriptionally represses NIS gene expression through the impairment of transcription factors Pax8 and p65 activity and that PI3K/Akt pathway activation by iodide-induced ROS production is involved in this process [176].

In a defined model of genetically susceptible hosts to autoimmune response, i.e., NOD.H2^h4^ mouse, excess dietary iodine leads to an increased immunogenicity of Tg and increased expression of ICAM-1 (a molecule participating in immune response) on thyroidal follicular cells. It has been documented that iodide excess increases the generation of ROS and ICAM-1 expression in cultures of NOD.H2^h4^ mouse thyrocytes, and that the antioxidant diphenyleneiodium, an inhibitor of NADPH oxidase, reduced both phenomena [177]. Such results indicate a role of oxidative stress in the pathogenesis of thyroid autoimmunity induced by exposure to excess iodine.

### In vivo studies

Regarding the effects of iodine excess on oxidative stress even more studies have been performed in in vivo conditions.

Although no parameters of oxidative stress were measured in this study, it is worth mentioning at the beginning of this section that iodide in vivo (300 µg KI/animal) inhibited the expression of TPO and NIS mRNAs in the dog (previously treated with goitrogens and perchlorate) thyroid after 48 h from KI treatment [178]. Also in another in vivo study it was observed that repeated treatment of rats with KI (1 mg/kg b.w.) within a few days resulted in the downregulation of genes involved in the synthesis and secretion of thyroid hormones, such as those encoding for NIS and monocarboxylate transporter 8 (MCT8; the most specific thyroid hormone transporter) followed by a delayed decrease of TPO gene expression together with pendrin (PDS; the apical iodide transporter in the thyroid contributing to iodide efflux) upregulation; at the same time, however, thyroid hormone level was not affected [179]. Unfortunately, parameters of oxidative stress were not measured in the above two studies [178, 179]. The above results [178, 179] are also illustrated in Fig. 2.

It should be noted that the issue regarding the order of events in the thyroid in response to iodine excess is not clearly defined. Differences in observations may result from different parameters measured (e.g., gene expression vs. enzyme activity vs. thyroid hormone level), models in vitro vs. in vivo, acute vs. chronic iodine treatment, etc. [107–117, 178, 179].

Iodine was shown to enhance oxidative processes not only in the thyroid gland [180–183] but also in various other tissues, in which different oxidative effects were possibly related to different sensitivity to iodine-induced oxidative damage [180–184]. Iodine-rich diet given to rats with normal thyroid function increased lipid peroxidation and catalase activity in the thyroid, the liver, and in the blood [180]. In another study, in response to iodine-rich diet administration the level of lipid peroxidation, measured as Schiff’s base concentration, increased in rat lungs and liver [181, 184]. Not only in euthyroid rats but also in rats with experimentally induced hypothyroidism, iodine treatment increased serum level of lipid peroxidation products and decreased NIS gene expression [183].

In another study, iodine-induced cytotoxicity and involution of the thyroid were caused by administering a twofold physiological dose of iodine in feeding water, which resulted in the increased level of 4-hydroxynonenal and 8-hydroxyguanine (markers of oxidative damage to membrane lipids and DNA, respectively) [185]. The amelioration of iodine-induced cytotoxicity was caused by cotreatment with antioxidant vitamin E [185].

Strongly enhanced oxidative stress, assessed by the increased formation of 4-hydroxynonenal, was found in thyroids when using a rat model of goiter formation (caused by iodine-deficient diet) and iodine-induced involution (caused, among others, by daily intraperitoneal injections of 100 μg iodide for 3 days); levels of glutathione peroxidases and peroxiredoxins (markers of antioxidant defense) were also upregulated in both groups, however, to lower extent in the latter [145]. This indicates that thyrocytes are well adapted to endogenously produced ROS in case of thyroid pathologies caused by either iodine deficiency or its excess.

Excess of iodine may also affect the process of spermatogenesis. It has been observed that chronic treatment with KI in rats results in the loss of spermatogenesis, decreased activities of enzymes participating in testosterone production, structural and functional changes of the testis, all these abnormalities being accompanied by the increased oxidative stress [186, 187].

Unfavorable effects resulting from iodine-induced generation of ROS can also include hyperglycemia, hypercholesterolemia, development of cardiovascular risk, renal degeneration, skeleton muscular disruption, degenerative changes [188], or hepatic steatosis [189]. Exposure to high iodine level may increase oxidative stress also in lymphocytes [190], which can contribute to the induction of autoimmune diseases.

As mentioned above, antioxidants play an important role by diminishing oxidative stress. In in vivo conditions melatonin [181], as well as PTU [184], prevented iodine-induced oxidative damage in lungs and liver, and also selenium revealed such a protection in the thyroid [191]. Interestingly, comparing to experimentally used iodine in excess, herbs with excess iodine damage rat thyroid follicular cells less, which may be related to high antioxidant capacity of these herbs [192]. An important position in protection against iodine-induced oxidative stress may have a nuclear factor erythroid 2-related factor 2 (Nrf2), being a transcription factor regulating the expression of some antioxidative enzymes, such as peroxiredoxins and sulfiredoxin, because both these enzymes were activated by iodine-rich diet [193].

It is worth pointing out that a single intake of high-dose KIO_3_, in contrast to chronic use [180, 183], does not affect total antioxidant activity, the parameter being frequently used to rapidly measure extracellular antioxidant defense [194]. It has been observed, however, that chronic mild and moderate iodine excess may weaken the antioxidative protection in the rat thyroid [195].

In pigs, which are considered tolerant concerning iodine excess, high iodine doses up to 10 mg/kg caused a significant downregulation of NIS in the thyroid gland and, simultaneously, it decreased mRNA expression of clue antioxidative enzymes, such as SOD and GPx, either in the liver or in the kidney or in muscles (target tissues of thyroid hormone action), however without affecting lipid peroxidation [196].

It is worth mentioning that iodoacetic acid (IAA), the most genotoxic iodinated disinfection byproduct known in drinking water, has recently been shown in in vitro and in vivo studies to act as a potential thyroid disruptor at various levels. Among others, exposure to IAA significantly reduced thyroid hormone-activated cell proliferation and significantly downregulated the TSH receptor and NIS at mRNA and protein levels [197]. More recently it has been suggested that IAA has reproductive and developmental toxicity, however with still unclear mechanisms [198].

### Studies in human subjects

There are not much available data from studies performed specially on purpose to evaluate the effects of excess iodine in humans.

The study performed in order to find the safe upper level of total daily iodine intake among Chinese adults revealed that subclinical hypothyroidism developed in initially euthyroid individuals who were taking for 4 weeks a daily dose of at least 400 μg iodine (as KI) as a supplement (which, together with a diet, provided a total iodine intake of approximately 800 μg/d) [199]. Instead, taking iodine supplements in a dose 100–300 μg/d did not modify thyroid function in a population with adequate iodine intake; however, it caused mild antioxidative action, as evaluated by the positive correlation between urinary iodine concentration or FT_4_ concentration and the level of antioxidative enzyme GSH-Px [200].

In the study performed on lactating women, who were taking 300 μg/d iodine (also in the form of KI), iodine level in breast milk correlated negatively with activities of antioxidative enzymes, such as SOD, GSH-Px, and catalase [201]. These results were confirmed in vitro—incubation of human adipocytes with 1 μM KI (which corresponds to the human breast milk iodine concentration) also caused a decreased expression of above antioxidative enzymes mRNA. The authors concluded that iodine may be involved in the regulation of oxidative stress in human breast milk [201]. It should be underlined that there is no contradiction between this association [201] and cited above results [200], as expression and activities of antioxidative enzymes can change differently depending on environment in which they are measured, time point, etc.

It has been shown in a recently published study that long-term exposure to high concentrations of iodine in drinking water, i.e., above 300 µg/L in non-pregnant adults and above 450 µg/L in pregnant and lactating women, was associated with an increased risk of abnormal blood pressure and abnormal blood glucose concentration, mainly in the latter group of individuals [202]. It is worth mentioning that in another study the same authors have shown favorable effects of normal iodine supply (comparing to iodine deficiency) on lipid profile. Drinking water with iodine concentration >100 µg/L (vs. 40–100 µg/L) was associated with a lower occurrence of hypertriglyceridemia and of high LDL-cholesterol, and a higher occurrence of high HDL-cholesterol in the adult population [203].

## Summary

According to The Endocrine Society definition of an EDC [1], iodine does fulfill the criteria of EDC because it is an exogenous chemical that interferes—when in excess—with thyroid hormone synthesis. Thus, it can, theoretically, join other either confirmed or potential thyroid disruptors [24, 25, 204]. Although iodine is not unequivocally classified as an EDC until now, it can be—comparing to certain classic EDCs—even more dangerous in some individuals exposed to supraphysiological doses of this micronutrient. Whereas legendary bisphenol A is broadly widespread in environment, resulting in universal exposure of populations to this EDC, and it is documented to be highly toxic under experimental conditions, generally it does not cause visible abnormalities in individuals. Instead, only some individuals can be exposed to iodine excess and still no abnormalities develop in most of them, but in a certain percentage the typical symptoms of hormone disruption (in this case—mostly hypo- or hyperthyroidism) are observed, sometimes being extremely dangerous, especially when the exposure to iodine excess occurs during fetal and early postpartum life.

Similar to typical EDCs [10], iodine does not have a precisely defined threshold dose above which it is definitely dangerous and below which it is absolutely safe in all exposed individuals.

If iodine is classified as EDC, the question arises what would be practical consequences regarding guidelines for iodine prophylaxis, other aspects of iodine use, etc.? That would not change general rules substantially regarding iodine supply, which means that iodine deficiency should be still eliminated worldwide and, at the same time, iodine excess should be avoided. However, universal awareness that iodine is an EDC would make consumers more careful regarding what they supplement in tablets and even what they eat and drink, etc., and—what is of great importance—it would make caregivers choose iodine-containing medications (or other medical products, such as used in diagnostics) more prudently. At the same time, however, it should be strongly stressed that no real iodine-dependent danger is associated with iodized salt consumption or additional iodine prophylaxis at preconception, during pregnancy and postpartum (also in hypothyroid women) and that to resign from such kinds of iodine prophylaxis would be not only unjustified but could be even harmful in consequence.

Besides the above-discussed most basic aspects of the assumption that “iodine is an EDC”, two others should be considered. First, it would help to identify other EDCs causing thyroid hormone disruption (acting, e.g., via blocking iodine action). Second, possible iodine-induced abnormalities should be also considered and further investigated in non-thyroidal tissues, especially those possessing NIS (although not equipped with complete machinery to synthesize thyroid hormones), for example, placenta or breast.

The assumption that oxidative stress is involved in hormone-disrupting properties of iodine has a separate practical value. It relies on the adherence to the principles regarding how to keep oxidative balance promoting health, i.e., via avoiding exogenous pro-oxidants in excess and consuming antioxidants in possibly optimal amounts, but also via avoiding such risk factors as obesity, low physical activity, etc., associated with the increased oxidative stress. In addition, it opens a new field on the role of oxidative stress as one of the mechanisms (if not the basic) of EDC action.

Whereas iodine excess may—under certain clinical condition—be associated with its disrupting action, adequate iodine supply, next to its main effect being the elimination of IDD, also reduces the risk of undesired actions of other EDCs in the thyroid. Taking into account that organisms are usually exposed at the same time to numerous EDCs, compliance with the rules concerning optimal iodine supply is of basic importance resulting in optimal thyroid hormone synthesis and in protection against EDCs (mostly those which act on the thyroid via NIS). The only exceptions from these rules relate to recommended large doses of stable (non-radioactive) iodine either to protect the thyroids of a given population exposed to radioiodine during a nuclear power plant accident [121] or in certain diagnostic and treatment procedures that are necessary for some individuals.

At the end of the review, it should be stressed that compared to iodine deficiency, iodine in excess (acting either as a potential EDC or via other mechanisms) is much less harmful in such a sense that it affects only a small percentage of exposed individuals who are sensitive, whereas the former affects whole populations; therefore, it causes endemic consequences.
